# Use of deep learning for the classification of hyperplastic lymph node and common subtypes of canine lymphomas: a preliminary study

**DOI:** 10.3389/fvets.2023.1309877

**Published:** 2024-01-12

**Authors:** Magdalena Hubbard-Perez, Andreea Luchian, Charles Milford, Lorenzo Ressel

**Affiliations:** DiMoLab, Institute of Infection Veterinary and Ecological Sciences, Department of Veterinary Anatomy Physiology and Pathology, University of Liverpool, Liverpool, United Kingdom

**Keywords:** lymphoma, canine, convolutional neural network, Artificial Intelligence, transfer learning

## Abstract

Artificial Intelligence has observed significant growth in its ability to classify different types of tumors in humans due to advancements in digital pathology technology. Among these tumors, lymphomas are quite common in dogs, despite studies on the application of AI in domestic species are scarce. This research aims to employ deep learning (DL) through convolutional neural networks (CNNs) to distinguish between normal lymph nodes and 3 WHO common subtypes of canine lymphomas. To train and validate the CNN, 1,530 high-resolution microscopic images derived from whole slide scans (WSIs) were used, including those of background areas, hyperplastic lymph nodes (*n* = 4), and three different lymphoma subtypes: diffuse large B cell lymphoma (DLBCL; *n* = 5), lymphoblastic (LBL; *n* = 5), and marginal zone lymphoma (MZL; *n* = 3). The CNN was able to correctly identify 456 images of the possible 457 test sets, achieving a maximum accuracy of 99.34%. The results of this study have demonstrated the feasibility of using deep learning to differentiate between hyperplastic lymph nodes and lymphomas, as well as to classify common WHO subtypes. Further research is required to explore the implications of these findings and validate the ability of the network to classify a broader range of lymphomas.

## Introduction

Lymphomas are a common type of neoplasm found in canines (1). They typically derive from lymphoid tissues, including lymph nodes, bone marrow, and spleen, although they can develop within any tissue in the body (2). Lymphomas can arise from both B- and T-cell lymphocytes, and the origin of the lymphoma often determines its form (2). B-cell lymphomas are the most common in canines, with approximately 65–75% being B cell and 25–35% being T cell (3). There are multiple forms of lymphomas, the most common being multicentric, with a prevalence of 84% in dogs (2). Other forms include alimentary, mediastinal, and cutaneous, which are much more uncommon (2). Canine lymphomas share many characteristics with human non-Hodgkin lymphomas (NHL) (4). Due to the similarity between human NHLs and canine lymphomas, the World Health Organization (WHO) system for human NHLs has been adapted to classify the types of canine lymphomas (4). Even though considered outdated by some authors, the updated Kiel classification is also an appropriate and commonly used classification for canine lymphomas (5). According to the WHO classification, three of the most diagnosed lymphomas are diffuse large B cell lymphoma (DLBCL), marginal zone lymphoma (MZL), and lymphoblastic lymphoma (LBL). Diffuse large B cell lymphoma (DLCBL) is the most common form of lymphoma found in dogs, with 79% of subjects being diagnosed with this subtype (3). Histologically, they are defined as having a diffuse pattern with uniformly large nuclei (6). These nuclei are generally round and infrequently cleaved or indented, with a variable mitotic rate (4). Dogs with this type of lymphoma commonly present with generalized lymphadenopathy, and they are typically classified at stages III to V using the WHO system (7). It is unclear if this type of lymphoma is more common in specific breeds. However, some studies indicate that Golden Retrievers, Labrador Retrievers, Bernese Mountain Dogs, and German Shepherds are more frequently affected (6). Marginal zone lymphomas (MZL) are the second most common lymphoma in dogs, with 17% of cases being classified as MZL (3). It is a B-cell lymphoma that is typically found in either the lymph nodes or the spleen (4). They are characterized as having a nodular pattern, with intermediate-sized cells with a central nucleolus and abundant lightly stained cytoplasm compared with other lymphomas (6). Mitoses are prominent only in advanced cases (4). This type of lymphoma is characterized morphologically by its “fading germinal centers,” which makes it resemble the marginal zone of a typical lymph node follicle (3). In addition to this, late stage MZL keep their cellular characteristics, but they lose their distinctive nodular pattern (6). This makes it challenging for pathologists to differentiate between this subtype and DLBCL (6). Dogs with this type of lymphoma generally present to the clinic with an enlarged submandibular or cervical lymph node which remains mobile under the skin. Most often, MZLs are present in large-breed dogs (4). Lymphoblastic lymphomas can originate from either B or T cell, although T phenotype is much more frequently found for this type (8). It is a less common lymphoma type if compared with DLBCL or MZL (9). Histologically, they are characterized by having uniformly intermediate-sized nuclei with evenly distributed chromatin, which conceals nuclear detail and a high mitotic rate (4). It is the most aggressive subtype frequently found in practices with dogs presenting with one or more enlarged peripheral lymphnodes (4). The study by Comazzi and Riondato (9) also suggested that this type of lymphoma is more frequently found in boxers. Due to their different morphological characteristics, these three common types of lymphoma represent a good target to explore the ability of Artificial Intelligence to discriminate between them and distinguish a lymphoid neoplastic proliferation (for which they offer common examples) from hyperplastic lymphoid tissue. In fact, reactive lymph node hyperplasia represents the main differential diagnosis for an enlarged lymph node. The most used approach for image classification is deep learning (DL) implemented with convolutional neural networks (CNNs) (10). Deep Learning is a form of machine learning where a neural network’s design is influenced by the human brain structure (11) and learns by example directly from the given data. Examples of types of data include images, sound, and text. This form of machine learning is known for achieving very high accuracies in image classification. These networks can achieve high accuracies if trained with a large amount of data and a deep network with multiple layers that allow learning millions of different features (11). Transfer learning is a common method used in DL. In this form of learning, a pre-trained network is selected and used as a starting point to train a network to learn a new task. Specific features are added or swapped out in order to alter the process before the training occurs. This allows the network to build from its previous knowledge and achieve the goal more successfully (12). This is one of the most used methods of training as it is easier to implement and is generally faster as it only involves fine-tuning (13). The use of machine learning and DL, in particular for processing and analyzing digital images in pathology, has become more popular in recent years. This is due to the rise in the use of whole digital slide scanners and an increased interest in digital pathology, allowing image analysis to be approached more freely (14). This project aims to investigate the feasibility of a CNN to distinguish hyperplastic lymph node from lymphoma and classify three common types of canine lymphoma. This is approached using a transfer learning strategy.

## Materials and methods

**Figure 1:**
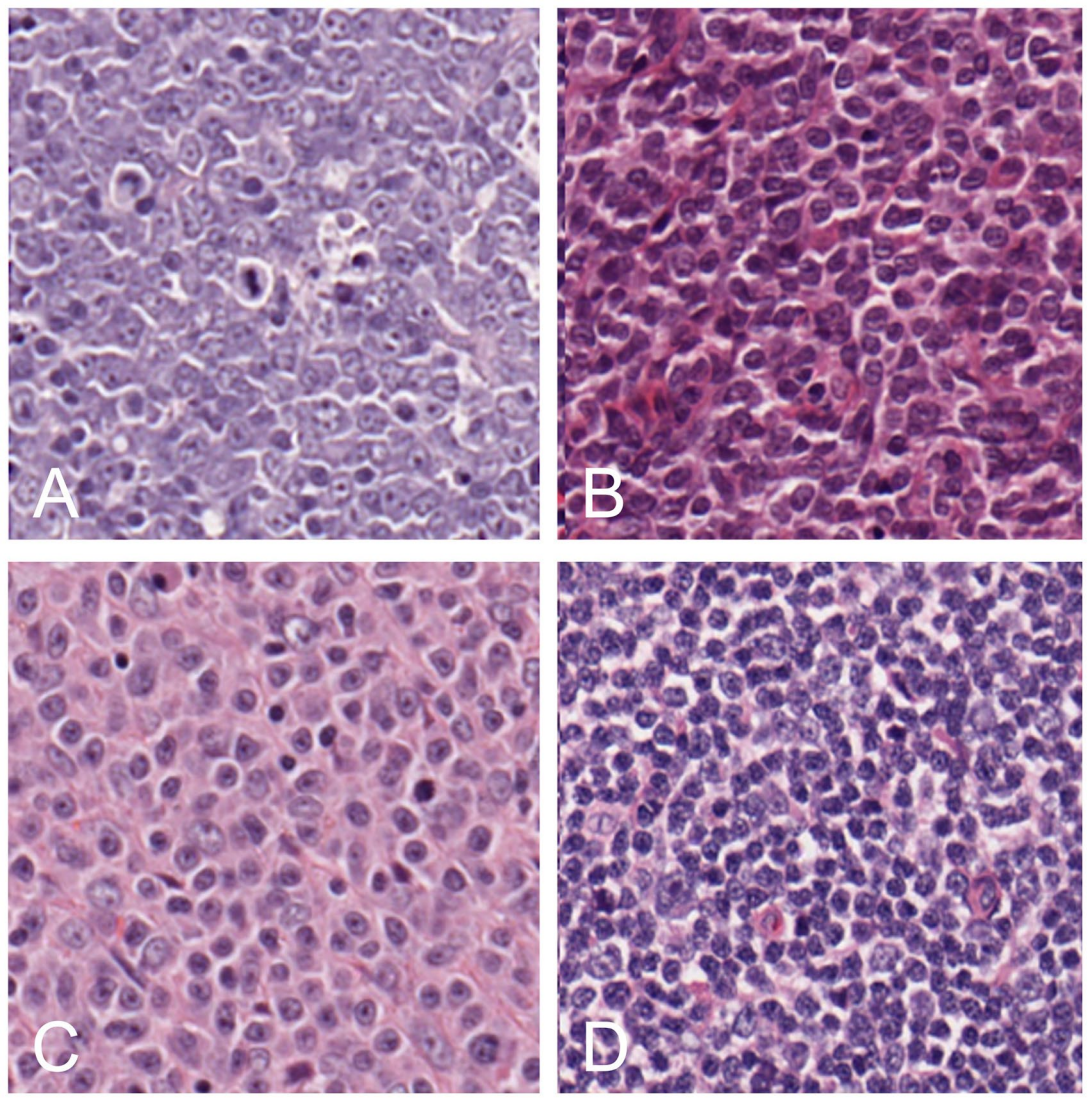
Examples of tiles of different classes: DLBCL **(A)**, LBL **(B)**, MZL **(C)**, and hyperplastic lymph node **(D)**.

**Figure 2:**
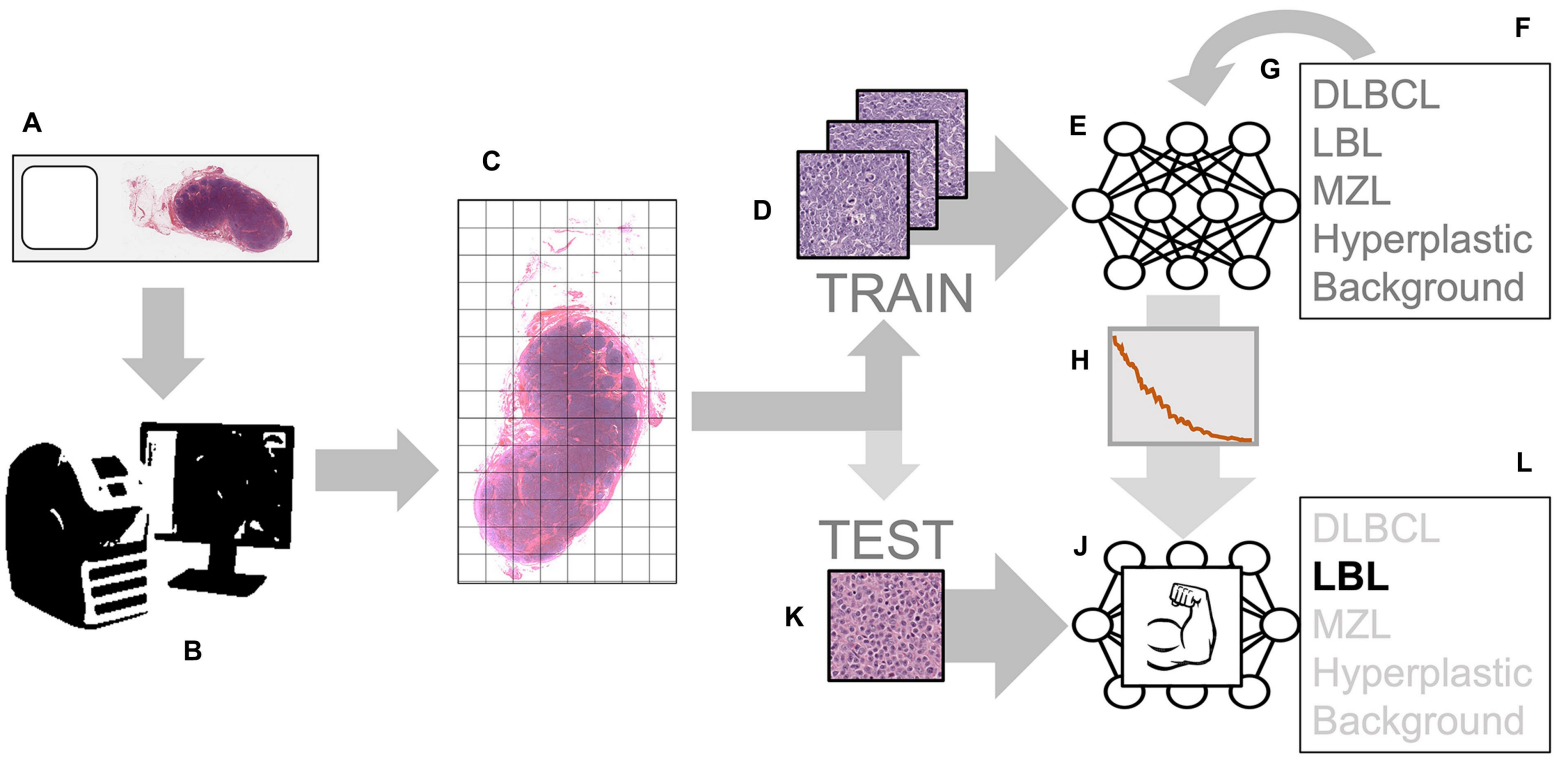
Workflow for training and testing the convolutional neural network (CNN): the process starts with histological slides representing various classes, such as DLBCL, LBL, MZL, and Hyperplastic lymph nodes **(A)**. These slides are scanned using a digital slide scanner, creating a Whole Slide Image (WSI) **(B)**. The WSI is divided into smaller tiles **(C)**. These tiles from different classes are used as examples to train the input layer of the CNN **(D)**. The CNN processes the input tiles **(E)** and predicts the probability of each class **(F)**. The error in these predictions is then used to adjust the neuron weights through back propagation **(G)**. This process continues until the error is minimized to an optimal value **(H)**. The result is a “trained” network **(J)**. This trained network can now be used to analyze never-before-seen tiles obtained through the same tiling process **(K)**. When fed with these new tiles, the network accurately predicts the class **(L)**.

Different cases of lymphoma and reactive hyperplastic lymph node slides were selected from the slides archive of the Department of Veterinary Anatomy Physiology and Pathology, Section of Veterinary Pathology at the University of Liverpool. In total, 4 cases of hyperplastic lymph node, 5 cases for each DLBCL and LBL, and 3 cases of MZL were selected as the classic examples of each category, following the criteria present in the WHO classification (4). All slides were previously stained with hematoxylin and eosin (HE) staining and diagnosed by board-certified pathologists with the use of immunohistochemistry for the determination of the phenotype. Slides were scanned by an Aperio CS2 (Leica Biosystems, Wetzlar, Germany) microscope slide scanner using native 40X magnification. In the preview of the slides, the region of interest (ROI) was cropped, and a final whole slide image (WSI) was generated. The image analysis software QuPath (15) was used to convert all the WSIs into the tiles that would later be selected for training the neural network. For each lymphoma slide, the area of lymphoma was manually annotated as “tumor,” while for reactive lymph node, all the lymph node areas (cortex and medulla) were annotated as “lymph node.” The automate tab, followed by the script editor, was selected, and a custom script was used, which allowed the creation of non-overlapping tiles and 512x512x3 pixels in size. This was repeated for each type of lymphoma, background areas of the slide, and hyperplastic lymph node slides. Examples of each classification were selected among the large number of tiles generated with this process (range: 2000–18,000 depending on the size of the sample). For each lymphoma, 75 tiles of the best examples of each DLBCL (Figure 1A), LBL (Figure 1B), MZL (Figure 1C), or hyperplastic lymph node (Figure 1D) were manually selected and cross-checked by a board-certified veterinary pathologist (LR), making sure that they would represent morphological criteria mentioned in the WHO classification. For the background, 15 tiles from each slide were also selected from areas without any tissue. These tiles from each slide were then grouped into their classes and put into folders, resulting in 5 folders of tiles, with a maximum of 375 tiles per class to be input into the neural network. The training was achieved in MATLAB (R2022a, Natick, Massachusetts: The MathWorks Inc.) with Deep Network Toolbox installed, and the learning method was transfer learning. GoogLeNet was chosen among possible CNNs as a fast network while retaining high depth and accuracy (19). The last fully connected layer was deleted and replaced by a new untrained fully connected layer, while the output size was changed to five classes (DLBCL, LBL, MZL, Hyperplastic, and Background). The WeightLearnRateFactor and BiasLearnRateFactor values were also increased to 2 to make the overall learning rate faster. All the classified tiles were imported into the network. Among 1,530 tiles, each class was split randomly between training set (70%) and testing set (30%), and a hold-out cross-validation strategy was applied, as the test set was considered a good statistical representation of the whole dataset. The network automatically resized the images to fit with the pre-trained network input layer-accepted format from 512x512x3 to an input size of 224x224x3 (downsampling). Tiles were randomized between training and test every time the network was run. In the training options tab, one of the three solvers (sgdm, adam, or rmsprop) was selected (a solver is the stochastic gradient descent algorithm, which evaluates the learning gradient and updates the network parameters using a portion of the training data during learning of a neural network to reduce loss) as well as an initial learning rate. Once the deep learning training process was completed, the results for the run were recorded. After reviewing and recording the results, the network was rerun with a combination of different solvers and/or initial learning rate values until a combination with high accuracy and low losses was achieved. Once this experiment was run multiple times and the best solver was identified, the data were run in a refining experiment using Bayesian optimization in order to find the best learning rate and get the highest accuracy. In this optimization phase, the solver sgdm was used, and since an initial learning rate of 0.0075 had high accuracy, the learning rate range was set between 0.00075 and 0.075. A maximum limit of 30 trials was selected beforehand. All 30 trials were run. After the optimization was completed, the final hyperparameters are shown in Supplementary Table S1. From this data, graphs showing the training and validation accuracies and losses were generated during the learning process. When the training was completed, the network produced a confusion matrix to show how well it learned the data and areas of errors (performance) on test images (30% of the tiles generated). Parameters such as False Positives, Sensitivity, False negatives, and Specificity for each class were calculated. All the experimental steps were run on a computationally efficient PC equipped with AMD Ryzen Threadripper PRO 3995WX CPU, 6x NVIDIA RTX 3090 GPUs, 1.00 TB of RAM, and Windows 10 Professional. A graphical summary of the workflow of the CNN for training and testing is presented in Figure 2.

## Results

The network was run three times for each solver, each with a different learning rate, to work out a rough range of what learning rate would have higher validation accuracy. From these experiments, sgdm solver was able to run with a higher learning rate (0.005) while still having a high validation accuracy of 99.34%. The average accuracy of three different experiments, with a random split of training and testing tiles using sgdm solver with different learning rates, was 97.01% (Supplementary Table S2). Following Bayesian optimization, 18 out of 30 trials were identified as the best, which had a learning rate of 0.0057 (Supplementary Figure S1). The training plots showed that the network converged approximately 50 iterations, reaching accuracy close to 100% for training and test sets before the 250th iteration (Supplementary Figure S2). The results of the test set (examples of DLBCL, LBL, MZL, normal lymph node, and background) presented in a confusion matrix (Table 1) showed that the CNN was able to correctly identify 456 images of the possible 457 of the test set. All the LBL, MZL, hyperplastic, and background tiles were correctly predicted, while 90 examples of DLBCL out of 91 (98.9) were correctly identified, with one example of DLBCL wrongly classified as hyperplastic lymph node. Considering the performance of distinguishing hyperplastic lymph node from lymphoma (DLBCL+LBL + MZL), all the predictions for lymphoma were correct, while a single prediction for hyperplastic lymph node out of 90 was misclassified as lymphoma.

**Table 1:** Confusion matrix for the 5 different classes.

							**FN**	**SE**
**True class**	Background	**76**					0.0	100.0
	**DLBCL**		**111**			1	0.9	99.1
	**LBL**			**112**			0.0	100.0
	**MZL**				**67**		0.0	100.0
	Hyperplastic					**90**	0.0	100.0
		Background	**DLBCL**	**LBL**	**MZL**	Hyperplastic		
		**Predicted class**						
	**FP**	0.0	0.0	0.0	0.0	1.1		
	**SP**	100.0	100.0	100.0	100.0	98.9		

**DLBCL**: Diffuse Large B cell Lymphoma; **LBL**: LymphoBlastic Lymphoma; **MZL**: Marginal Zone Lymphoma; **FN**: False negatives; **SE**: Sensitivity; **FP**=False Positives; **SP**=Specificity. FN,SE,FP,SP parameters are expressed as percentages.

## Discussion

The present preliminary work demonstrated that transfer learning applied to the problem of differentiation between hyperplastic and neoplastic lymph node and of classification of lymphoid neoplasms in dogs can be successful. We also demonstrated that our methodological strategy for network learning hyperparameter optimization is promising. Our approach used a hold-out cross-validation approach, as the test set was considered a good statistical representation of the whole dataset. Given the average accuracy of 97.01% in 3 different experiments with different learning rates and randomization of training and test tiles, the network seems to perform consistently. Despite the study being preliminary and focusing primarily on the proof of principle of technique application, at present, there are no studies published applying CNNs to canine lymphomas, while machine learning has been applied only marginally to clinical data in canine lymphomas (16), with no exploration of the potential of CNNs. The accuracy of the network was very high. In the one image where the classification of the network was incorrect, it mistakenly categorized a DLBCL tile as a hyperplastic lymph node tile instead. This is encouraging, as the network was still able to correctly classify 99.1% of the DLBCL tiles presented to it, and it can be argued that a very high-power view of a germinal center in a hyperplastic node can overlap with a small area of DLBCL. Considered within a scenario where the outcome of multiple tiles should achieve the final diagnosis, this error can be considered almost irrelevant. It remains likely a challenge to establish, at tile level, what should be considered as normal lymph node, due to the heterogeneity of the lymph node structure, and likely a segmentation approach at lower magnification, similar to the one we recently approached on a similarly complex anatomical structure (kidney) (17) could prove an effective alternative strategy. However, it is interesting to observe that the network seems to capture features of different classes without the need for a sub-microscopic structure of the lymph node, likely relying on cytological features. One limitation of this preliminary study is, inherently, the low number of slides used in the experiments, which may not simulate fully the complexity of a wider scale experiment using a larger number of cases. However, the suggestion that features are learned at this accuracy rate using a reasonably low number of examples (hundreds per class) in the context of a transfer learning approach is promising for future larger experiments, which should include a larger and more diverse set of training and test datasets. In an ideal scenario, the development of a comprehensive CNN for facilitating AI-assisted diagnosis on a large scale would also gain significant advantages through the incorporation of diverse slide scanners and staining protocols sourced from various laboratories. It is important to note that, at present, this aspect remains one of the foremost limitations in the context of histology CNN approaches (18). The results from this experiment have shown that the GoogLeNet network is a highly reliable network to use for deep learning when applied to lymphoma histological images using a transfer learning approach. This strongly suggests that the basic patterns learned in the early layers during the original training on 1.2 million images and 1,000 different classes, representative of common objects and living beings (19), represent a solid starting point to refine the features for histological images of canine lymphomas. A smaller number of tiles were used for one of the neoplasm categories (MZL). However, despite this, there was no effect on the performance, suggesting that lymphomas are, in the “eyes” of the CNN, a reasonably homogeneous type of tumors, where a relatively low number of examples can result in successful training. There are many studies that discuss the similarities between canine and human lymphomas and how canine models may contribute to lymphoma research. Due to the similarities and use of a derived classification system, this preliminary result appears likely translational to human medicine. DLBCL lymphomas in both humans and canines are especially similar, as they resemble each other in many features, including that they are the most common subtype of B cell lymphoma in both dogs and humans (6). The workflow proposed uses easily accessible software: MATLAB and QuPath are both open-source software or offer free versions, making them accessible to researchers with limited budgets. Moreover, this software is commonly used in academic settings and has a wide base of users, which promotes transparency, reproducibility, and collaboration within the scientific community. In conclusion, this study has successfully shown that a convolutional neural network can be used to differentiate between hyperplastic and neoplastic lymph nodes, as well as classify specific subtypes of canine lymphomas. The integration of AI-aided diagnosis in canine lymphoma histology, among other areas, promises to revolutionize veterinary diagnostics, but potential benefits and challenges we might encounter as this technology becomes more prevalent are still to be unraveled; therefore further studies are encouraged. These findings not only demonstrate the potential of deep learning in distinguishing canine lymphomas from normal lymph nodes but also lay the groundwork for larger scale studies encompassing different species and a broader range of lymphoma subtypes.

## Data availability statement

The raw data supporting the conclusions of this article can be made available by the authors, without undue reservation upon request.

## Ethics statement

The animal study was approved by the School of Veterinary Science, University of Liverpool, Ethics Committee. The study was conducted in accordance with the local legislation and institutional requirements (RETH000942).

## Author contributions

MH-P: Conceptualization, Data curation, Investigation, Visualization, Writing – original draft, Writing – review & editing. AL: Conceptualization, Investigation, Methodology, Writing – review & editing. CM: Conceptualization, Investigation, Writing – review & editing. LR: Conceptualization, Project administration, Resources, Supervision, Visualization, Writing – review & editing.

## Supplementary material

Supplementary Figure S1 Screenshot of the outcome of the Bayesan optimization, with the chosen experiment 18 highlighted.

Supplementary Figure S2 Accuracy (blue) and Loss (red) curves for run 18: the network reached an optimal training due to high accuracy and very low loss.

Supplementary TABLE S1 Hyperparameters after optimization.

Supplementary TABLE S2 Validation accuracy running 3 experiments using 3 different solvers.
