# Supplementary figures and images for: Use of deep learning for the classification of hyperplastic lymph node and common subtypes of canine lymphomas: a preliminary study
Source: Front Vet Sci. 2024 Jan 12;10:1309877. doi: 10.3389/fvets.2023.1309877 (PMC10811236; doi:10.3389/fvets.2023.1309877)

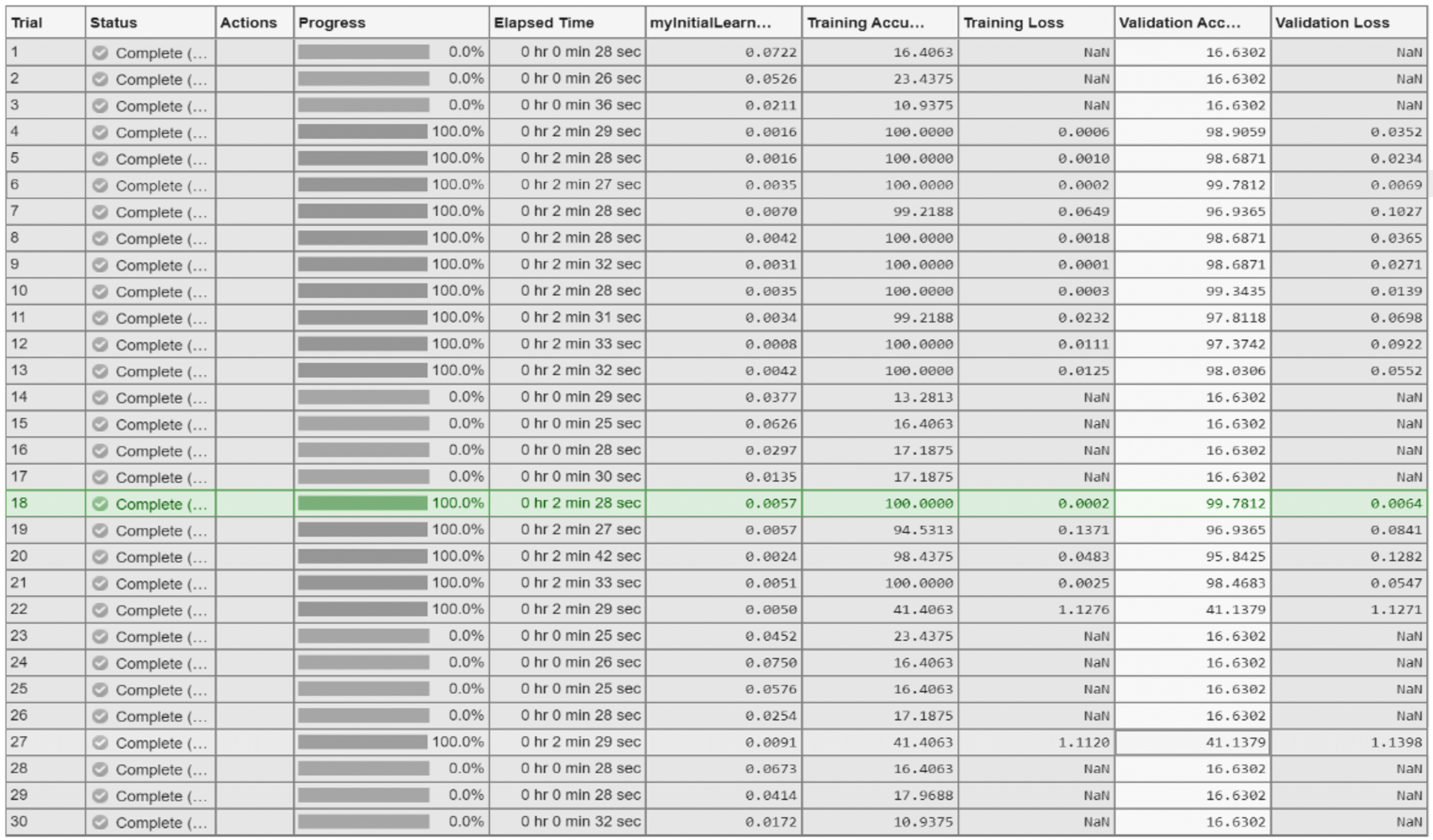

Supplement: Supplementary Figure S1 — Screenshot of the outcome of the Bayesan optimization, with the chosen experiment 18 highlighted. [file Image_1.JPEG]

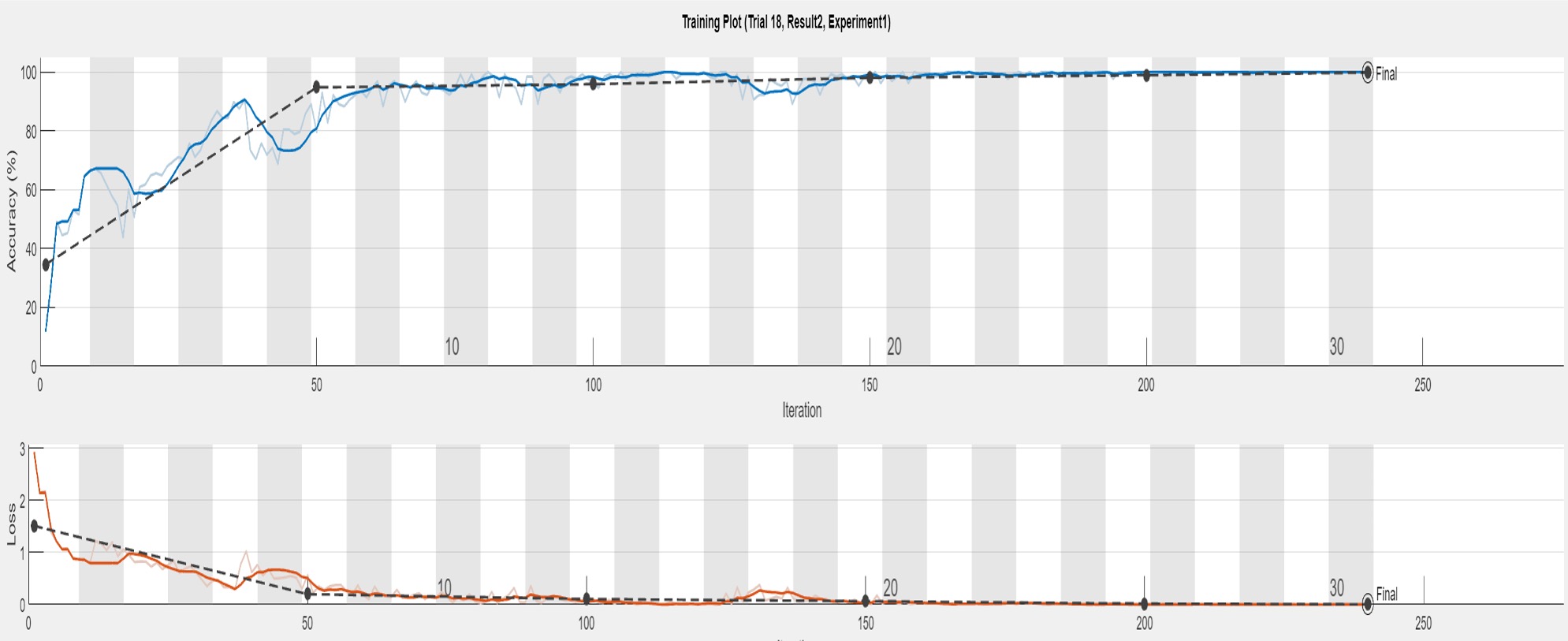

Supplement: Supplementary Figure S2 — Accuracy (blue) and Loss (red) curves for run 18: the network reached an optimal training due to high accuracy and very low loss. [file Image_2.JPEG]
